# Functional Analysis of *OsMED16* and *OsMED25* in Response to Biotic and Abiotic Stresses in Rice

**DOI:** 10.3389/fpls.2021.652453

**Published:** 2021-03-31

**Authors:** Huijuan Zhang, Dewei Zheng, Longfei Yin, Fengming Song, Ming Jiang

**Affiliations:** ^1^College of Life Science, Taizhou University, Taizhou, China; ^2^National Key Laboratory of Rice Biology, Institute of Biotechnology, Zhejiang University, Hangzhou, China

**Keywords:** MED, resistance, *M. grisea*, cold, VIGS

## Abstract

Mediator complex is a multiprotein complex that regulates RNA polymerase II-mediated transcription. Moreover, it functions in several signaling pathways, including those involved in response to biotic and abiotic stresses. We used virus-induced gene silencing (VIGS) to study the functions of two genes, namely *OsMED16* and *OsMED25* in response to biotic and abiotic stresses in rice. Both genes were differentially induced by *Magnaporthe grisea* (*M. grisea*), the causative agent of blast disease, hormone treatment, and abiotic stress. We found that both BMV: OsMED16- and BMV: OsMED25-infiltrated seedlings reduced the resistance to *M. grisea* by regulating the accumulation of H_2_O_2_ and expression of defense-related genes. Furthermore, BMV: OsMED16-infiltrated seedlings decreased the tolerance to cold by increasing the malondialdehyde (MDA) content and reducing the expression of cold-responsive genes.

## Introduction

Mediator complex, a multiprotein complex, was first discovered in yeast as a cofactor of RNA polymerase II that participates in RNA polymerase II-mediated transcription (Conaway and Conaway, 2011). Bioinformatics analysis and wet experiments revealed it to be a conserved multiprotein complex containing approximately 20 to 30 subunits. Although the number of these subunits vary in different organisms, they all form a four-module complex consisting of the head domain, middle domain, tail domain, and a cyclin-dependent kinase domain (Bourbon, 2008). Interestingly, the mediator complex is not stationary and uses isomerism to transfer numerous signals (Conaway and Conaway, 2011). Mediator complex was first purified from plants in 2007 (Backstrom et al., 2007). The sequences of its subunits in plants are considerably different from those in other eukaryotic organisms; however, their secondary structure is similar. For example, out of the 27 subunits present in the *Arabidopsis* mediator complex, 21 are conserved across eukaryotic organisms, whereas the remaining six are specific to plants (Backstrom et al., 2007; Kidd et al., 2011; Mathur et al., 2011). Recently, 28 conserved MEDs were identified in *Arabidopsis* including HAC1 and HAC5 (Guo J. et al., 2020). The mediator complex functions in several signaling pathways involved in development, response to biotic and abiotic stresses, and cellular movement (Samanta and Thakur, 2015; Malik et al., 2017; Chong et al., 2020a,b; Crawford et al., 2020).

MED8, MED12, MED13, MED14, MED15, MED16, MED17, MED18, MED19, MED20, MED21, MED25, CDK8, and MED37 have been implicated in the development (Autran et al., 2002; Lalanne et al., 2004; Wang and Chen, 2004; Gillmor et al., 2010; Ito et al., 2011; Xu and Li, 2011; Canet et al., 2012; Zheng et al., 2013; Lai et al., 2014; Hasan et al., 2020; Sun et al., 2020; Zhang and Guo, 2020; Agrawal et al., 2021). A study reported that a mutation in MED18 resulted in dwarfism, delayed formation of floral organs, late flowering, and reduced fertility (Kim et al., 2011). The underlying mechanisms of the function of MEDs in the development are being studied. MED16 regulates iron homeostasis by modulating iron uptake and gene expression and by associating with EIN3/EIL1 through the MED25 subunit (Yang et al., 2014; Zhang et al., 2014). MED8 and MED25 affect the size of plant organs by regulating cell proliferation (Lalanne et al., 2004; Xu and Li, 2011). MED18 affects flowering time and the formation of floral organs by regulating the expression of Flowering Locus C (FLC) and AGAMOUS (AG) (Zheng et al., 2013; Lai et al., 2014).

Mediator complex subunits CDK8, MED7, MED14, MED16, MED17, MED25, and MED36a are involved in response to abiotic stresses (Knight et al., 2009; Hemsley et al., 2014; Zhang et al., 2017; Ohama et al., 2021). CDK8 regulates abscisic acid (ABA) signaling and drought response in *Arabidopsis* by associating with RAP2.6, and SnRK2.6 (Zhu et al., 2020). MED16 regulates the response to cold stress, MED25 regulates the response to salt and drought stress (Boyce et al., 2003; Elfving et al., 2011). Furthermore, subunits MED8, MED14, MED15, MED16, MED18, MED19a, MED20, MED21, MED25, and MED36a are involved in response to biotic stress. The *med8* mutants, *med18* mutants, and MED21-silencing plants reduced the resistance to both *Alternaria brassicicola* (*A. brassicicola)* and *Botrytis cinerea* (*B. cinerea*) in *Arabidopsis* (Dhawan et al., 2009; Kidd et al., 2009; Lai et al., 2014). In addition, *Arabidopsis med8* mutants increased the resistance to *Fusarium oxysporum* (*F. oxysporum*) and *Pseudomonas syringae pv. tomato* DC3000 (*Pst* DC3000). *Arabidopsis med18* mutants increased the resistance to RNA and DNA viral infections (Kidd et al., 2009; Hussein et al., 2020). The *Arabidopsis* mutant *med25* decreased the resistance to *A. brassicicola* and *B. cinerea*, whereas it increased the resistance to *F. oxysporum* (Kidd et al., 2009). The mutants *med14* and *med15* decreased the resistance to *Pst* DC3000 in *Arabidopsis* (Canet et al., 2012; Zhang et al., 2013). In wheat, the MED15 mutant suppressed the resistance to stem rust (Hiebert et al., 2020). However, in *Arabidopsis*, *med16* mutants displayed decreased resistance to *Pst* DC3000/avrRpt2, *Pst* DC3000, *A. brassicicola*, *B. cinerea*, and *Sclerotinia sclerotiorum* (*S. sclerotiorum*) (Wathugala et al., 2012; Zhang et al., 2012; Wang et al., 2015). MED19a positively regulate the resistance to *Hyaloperonospora arabidopsidis* (Caillaud et al., 2013).

Our team focus on the functions of MEDs in plants such as tomato and rice. MEDs were found to have functions in the resistance to *Botrytis cinerea* in tomato, including MED16 and MED25. The silencing of MED16 or MED25 strongly decreased the resistance to *Botrytis cinerea* in tomato. However, MED16 or MED25 do not have functions on the tolerance to cold and drought stresses in tomato. Whether MED16 or MED25 have functions in responses to biotic and abiotic stresses in rice were studied in this study. In this study, we studied the functions of two genes, *OsMED16* (*Os10 g35560*) and *OsMED25* (*Os09 g13610*) in rice. The expression of *OsMED16* and *OsMED25* was induced by pathogen inoculation, hormone treatment, and stresses. The higher susceptibility of BMV:OsMED16- and BMV:OsMED25-infiltrated plants to *M. grisea*, when compared with the control, could be attributed to regulation of H_2_O_2_ accumulation and expression of defense-related genes. BMV: OsMED16-infiltrated plants decreased the tolerance to cold stress by regulating the MDA content and the expression of cold-responsive genes.

## Materials and Methods

### Characterization of OsMED16 and OsMED25

Rice genome database was searched using BlastP program with *Arabidopsis* AtMED16 and AtMED25 as queries, and the predicted nucleotide and amino acid sequences for *OsMED16* and *OsMED25* were downloaded. Phylogenetic trees for tomato, *Arabidopsis*, rice, and other MEDs were constructed using the Neighbor-joining method by MEGA6 program with the *p*-distance, complete deletion, and 1000 bootstraps.

### Plant Growth Conditions and Different Treatments

Yuanfengzao, a pair of isogenic lines (H8R and H8S), and IR64 were used for different analyses. For hormone treatment, 1.5 mM SA (salicylic acid, pH 6.5), 100 μM JA (jasmonic acid), 100 μM ACC (1-amino cyclopropane-1-carboxylic acid), or 100 μM ABA was sprayed on the leaves of 4-week-old Yuanfengzao seedlings. The same volume of water or 0.1% ethanol was used as control. Three-week-old plants were placed at 42°C for expression analysis of response to heat and at 4°C for cold. For drought treatment, the hydroponic 3-week-old plants were placed on the floor of the frame after the water was dried by filter paper. For NaCl stress, the hydroponic 3-week-old plants were treated with 200 mM NaCl solution. Samples were collected at specific time points for gene expression. Roots, flowers, stems, coats, leaves, sheaths, and ligules were collected for gene expression analysis in different tissues.

To infect the plants with *M. grisea* (strain 85–14B1, race ZB1), H8R and H8S lines were sprayed with spore solution as described earlier (Luo et al., 2005). IR64 was used for virus-induced gene silencing (VIGS) assays. A month later, silenced plants were used for different analyses. In every experiment, the samples were collected at indicated time points and stored at −80°C until use. All plants were kept in a room at 26°C, with a cycle of 14 h light (>3000 lux)/10 h dark, except those for VIGS assay, which were kept at 24°C, with a cycle of 14 h light (>3000 lux)/10 h dark.

### Vector Construction and VIGS Infiltration

PCR fragments (200–400) (sequences were showed in Supplementary Material) were digested by *Avr*II and *Nco*I, then ligated to modified BMV vector. The recombinant plasmids confirmed by sequencing were electroporated into *Agrobacterium tumefaciens* strain C58C1; the agrobacteria were used for VIGS infiltration as described earlier (Sun et al., 2013). Briefly, agrobacteria carrying recombinant plasmids were cultured in liquid YEP medium with antibiotics at 28°C in a shaker incubator overnight. The cells were collected by centrifugation and resuspended in an induction buffer. Next, the solution was kept at room temperature for 5 h, centrifuged, resuspended in infiltration solution, and kept at room temperature. Infiltration was stopped until the OD value reached 2.0. It was subsequently mixed with the same volume of *Agrobacterium* harboring pC13/F1 + 2 before vacuum infiltration. The aerial parts of 10-day-old IR64 seedlings were mixed with *Agrobacterium* suspension for 7 min at 20 Kpa. The obtained plants were recorded as BMV:OsMED16-infiltrated plants and BMV:OsMED25-infiltrated plants. A group of rice seedlings were infiltrated with agrobacteria harboring a construct of BMV:OsPDS (*Phytoene desaturase*) and used as positive controls for silencing evaluation of the VIGS procedure (data showed in Supplementary Material). Plants transfected with empty vector were used as controls, which were recorded as BMV:00-infiltrated plants.

### qRT-PCR Analysis of Gene Expression

RNA was extracted with TRIzol (Invitrogen, Shanghai, China) according to the manufacturer’s instructions. First-strand cDNA was synthesized by AMV reverse transcriptase (TaKaRa, Dalian, China). The qPCR was performed in a CFX96 real-time PCR detection system (Bio-Rad, Hercules, CA, United States) with a reaction volume of 25 μL containing 12.5 μL 2 × SYBR Premix Ex Taq^TM^ (TaKaRa, Dalian, China), 0.1 μg of cDNA, and 7.5 pmol of each gene-specific primer (see Table 1).

**TABLE 1:** The list of primer sequences used in this study.

**Primers**	**Sequences (5′-3′)**
*MED16*-qRT-F	CCTGCTGAAGAATGGCATAGA
*MED16*-qRT-R	CGATAAGTGGCGATTGGTAGAG
*MED25*-qRT-F	GAGAAGATCGTGCGGAGTTT
*MED25*-qRT-R	CGCTATAAGGACCATGGGTATG
*OsActin*-qRT-F	A AGCTGCGGGTATCCATGAGA
*OsActin*-qRT-R	GCAATGCCAGGGAACATAGTG
*MED16*-vigs-F	ATACCTAGG TGTCAGGAATTTCTCCGTAT
*MED16*-vigs-R	TATCCATGG TCACCTCCCAAACCACTA
*MED25*-vigs-F	ATACCTAGG ATACAATGCGGCAAAGAG
*MED25*-vigs-R	TATCCATGG AAATAAGGGACGGTGAGG
eEF1-qRT-F	CAACCCTGACAAGATTCCCT
eEF1-qRT-R	AGTCAAGGTTGGTGGACCTC
28s rDNA-qRT-F	TACGAGAGGAACCGCTCATTCAGATAATTA
28s rDNA-qRT-R	TCAGCAGATCGTAACGATAAAGCTACTC
*OsLOX1*-qRT-F	AAACGCTCGCTGGCATCAAC
*OsLOX1*-qRT-R	ATCGCCTCCTCCACCGTCAT
*OsNH1*-qRT-F	GCGGCGTCTCCTTGATGTCCTT
*OsNH1*-qRT-R	CGAGTTGTGGGTCCCTTCTTTC
*OsPR1a*-qRT-F	TCGTATGCTATGCTACGTGTTT
*OsPR1a*-qRT-R	CACTAAGCAAATACGGCTGACA
*OsPR3*-qRT-F	CACATACTGCGAGCCCAA
*OsPR3*-qRT-R	TTGTAGGTGATCTGGATGGG
*OsWRKY45*-qRT-F	CGGGCAGAAGGAGATCCAAAACT
*OsWRKY45*-qRT-R	GCCGATGTAGGTGACCCTGTAGC
*Myb*-qRT-F	ACGGCGGTGGGATTTCTTA
*Myb*-qRT-R	GCGATGCGAGACCACCTGTT
*CDPK7*-qRT-F	AACATGCCCGATGCTTTTCTT
*CDPK*-qRT-R	ATTGTTCTTCGTCCGACTCCC
*Fer1*-qRT-F	GGGAAAGGGAAGGAGGTGCT
*Fer1*-qRT-R	GTAGGCGAAAAGGGAGTGGT
*Trx23*-qRT-F	GTTCCCTGGTGCTGTCTTCC
*Trx23*-qRT-R	GCTTCACGATGGTGTTCTGG
*Lti6a*-qRT-F	CGGCGTCTTCTTCAAGTTCG
*Lti6a*-qRT-R	TGAGCAGCAAGCAGATCCAG

### Fungal Culture and Disease Assay

*Magnaporthe grisea* was cultivated on oatmeal agar for 15 days until spores covered the whole surface. Next, the spores were washed to prepare a solution. For detached leaf analysis, the completely expanded leaves were obtained from 4-week-old plants and placed on a wet filter paper. Next, 5 μL of spore suspension was dropped on the surface of leaves. For whole-plant analysis, the plants were sprayed with the spore solution. Afterward, the tray was covered with a preservative film and kept in the room used for silencing plant growth. The images were acquired 7 days later, the lesion size was recorded, and the fungal growth was measured.

### Cold Stress Assay and Physiological and Biochemical Measurements

For cold stress assay, 4-week-old BMV:OsMED16-infiltrated plants and BMV:00-infiltrated plants (control) were grown in the same pot at 4°C for 1 day, then transferred to normal conditions for recovery. Plants with more than 20% green leaves were considered to have survived, and the others were considered dead. The survival rate was calculated as the percentage of survival among total plants. The relative electrolyte leakage, chlorophyll content, malondialdehyde (MDA) content, H_2_O_2_ content, SOD activity, and CAT activity were measured as described previously (Lichtenthaler, 1987; Mittova et al., 2000; Alexieva et al., 2011; Song et al., 2011). *In situ* detection of H_2_O_2_ in leaf tissues was performed by DAB staining (Thordal-Christensen et al., 1997).

## Results

### Characterization of OsMED16 and OsMED25 in Rice

**FIGURE 1:**
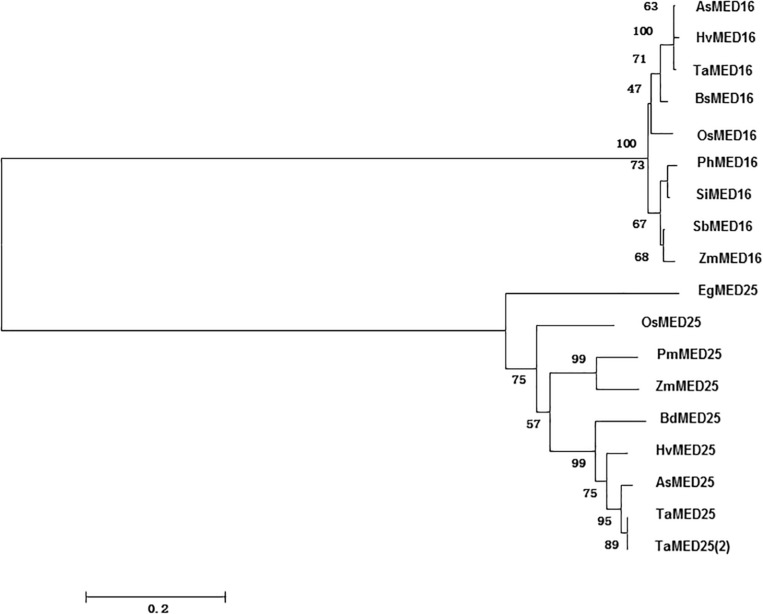
Phylogenetic tree of OsMED16 and OsMED25. Phylogenetic tree was constructed by neighbor-joining method using MEGA program version 6.0.

By BlastP searches against the rice genome database using the characterized *Arabidopsis* AtMED16 and AtMED25 as queries, OsMED16 and OsMED25 were obtained. Phylogenetic tree analysis revealed that OsMED16 and OsMED25 showed more than 40% of sequence identity to *Arabidopsis* AtMED16 and AtMED25 which were already reported to have functions in response to biotic and abiotic stresses (Figure 1).

### The Expression of *OsMED16* and *OsMED25* Was Induced by Different Stimuli

**FIGURE 2:**
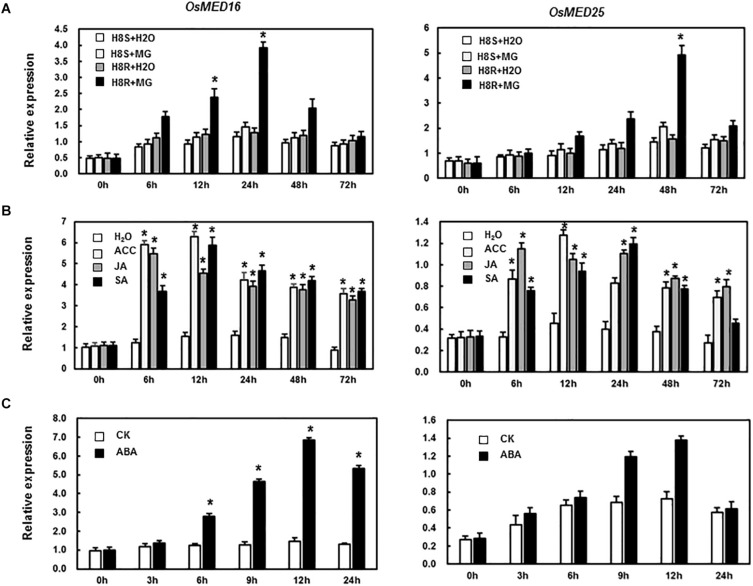
Expression patterns of *OsMED16* and *OsMED25* genes in response to *Magnaporthe grisea* infection and hormone treatment. **(A)** The expression patterns of *OsMED16* and *OsMED25* following inoculation with *M. grisea.* **(B)** The expression patterns of *OsMED16* and *OsMED25* following treatment with 1.5 mM SA, 100 μM JA, and 100 μM ACC. **(C)** The expression patterns of *OsMED16* and *OsMED25* following treatment with 100 μM ABA. The data were normalized against *OsActin* gene, and the relative expression was shown as fold expression of *OsActin*. Data are presented as means ± SD from three independent experiments and * above the columns indicate significant differences at *p* < 0.05 between the pathogen-inoculated or hormone-treated plants and the mock-inoculated/treated plants.

To study if *OsMED16* or *OsMED25* contributed to disease resistance, we first analyzed the responsiveness of these two genes in rice after pathogen infection and treatment with defense-related signal molecules. As shown in Figure 2A, the expression of *OsMED16* and *OsMED25* increased significantly in incompatible interaction but was different from each other. The expression of *OsMED16* was the highest in the incompatible interaction after 24 h, whereas that of *OsMED25* was highest in the incompatible interaction after 48 h. In the treatment of ACC, JA, and SA, *OsMED16* and *OsMED25* were induced significantly by all three signal molecules (Figure 2B).

**FIGURE 3:**
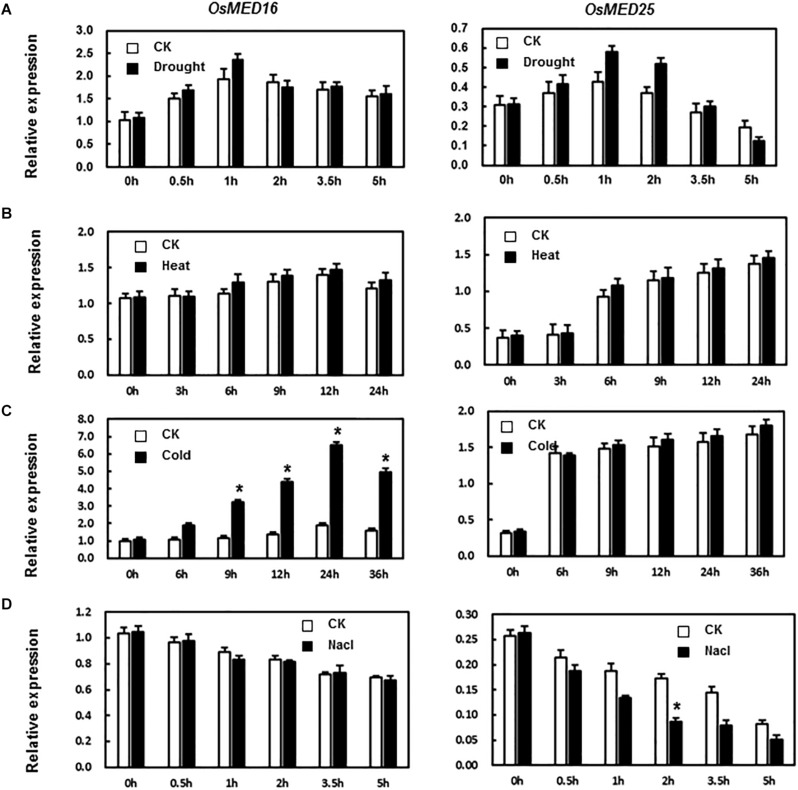
The expression patterns of *OsMED16* and *OsMED25* genes in response to drought, heat, cold, and NaCl stress. **(A)** The expression patterns of *OsMED16* and *OsMED25* under drought stress. **(B)** The expression patterns of *OsMED16* and *OsMED25* under heat stress. **(C)** The expression patterns of *OsMED16* and *OsMED25* under cold stress. **(D)** The expression patterns of *OsMED16* and *OsMED25* under NaCl stress. The samples were collected at different time points for analysis of gene expression by qRT-PCR. Expression data were normalized against *OsActin* gene as the reference, and the relative expression is shown as fold expression of *OsActin*. Data are presented as means ± SD from three independent experiments, and * above the columns indicate significant differences at *p* < 0.05 between the treated plants and the control.

MED16 and MED25 were reported to differentially regulate ABA signaling in *Arabidopsis* (Guo P. C. et al., 2020). So we tested whether the expression levels of *OsMED16* or *OsMED25* were induced by ABA treatment in rice. As shown in Figure 2C, after treatment with ABA, the expression of *OsMED16* increased dramatically, whereas that of *OsMED25* showed no significant difference as compared with the control. To study whether *OsMED16* or *OsMED25* responded to abiotic stresses, we analyzed the responsiveness of these two genes in rice under abiotic stresses. In the drought and heat stresses, the expression of *OsMED16* and *OsMED25* showed no significant difference as compared with the control (Figures 3A,B). In cold stress, the expression of *OsMED16* was induced dramatically, whereas that of *OsMED25* showed no significant difference as compared with the control (Figure 3C). In salt stress, the expression of *OsMED25* decreased slightly, whereas *OsMED16* showed no significant difference when compared with the control (Figure 3D).

**FIGURE 4:**
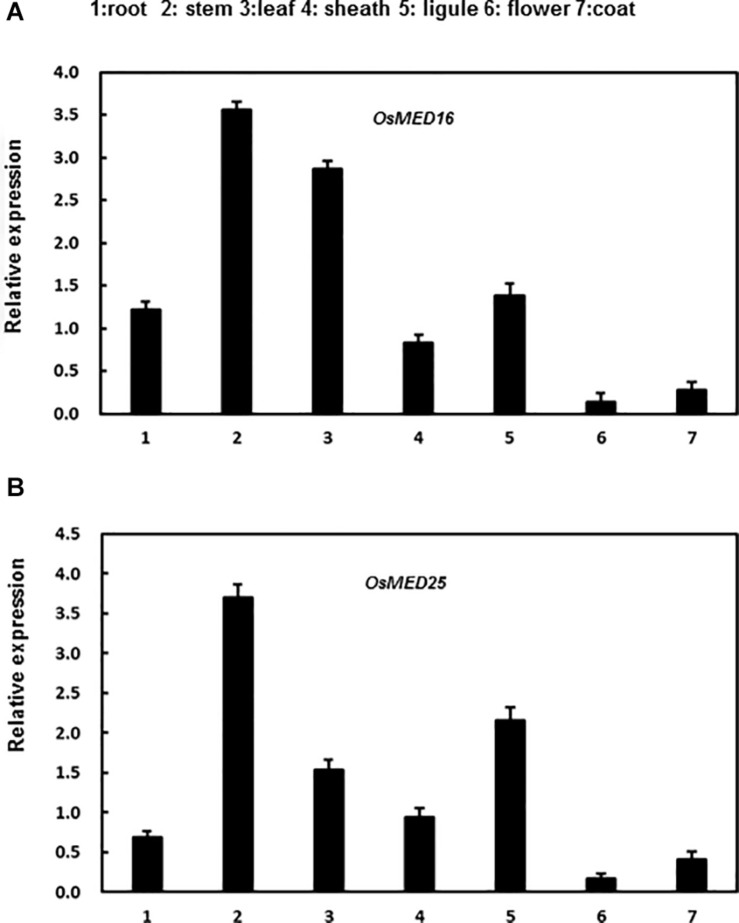
The expression patterns of *OsMED16* and *OsMED25* genes in different tissues. **(A)** The expression patter of *OsMED16* in different tissues. **(B)** The expression patter of *OsMED25* in different tissues. Roots, flowers, stems, coats, leaves, sheaths, and ligules were collected to analyze the gene expression by qRT-PCR. Expression data were normalized against *OsActin* gene, and the relative expression is shown as fold expression of *OsActin*. Data are presented as means ± SD from three independent experiments.

In addition, we analyzed the expression patterns of these genes in various tissues of rice seedlings. Both genes were expressed in all tissues assessed to varying levels in different tissues. *OsMED16* was highly expressed in roots, stems, leaves, and ligules; however, the expression was low in flowers, sheaths, and coats (Figure 4A). Moreover, *OsMED25* was highly expressed in the stems, leaves, sheaths, and ligules; however, the expression was low in roots, flowers, and coats (Figure 4B).

### *OsMED16* and *OsMED25* Silencing Did Not Affect Growth and Development

**FIGURE 5:**
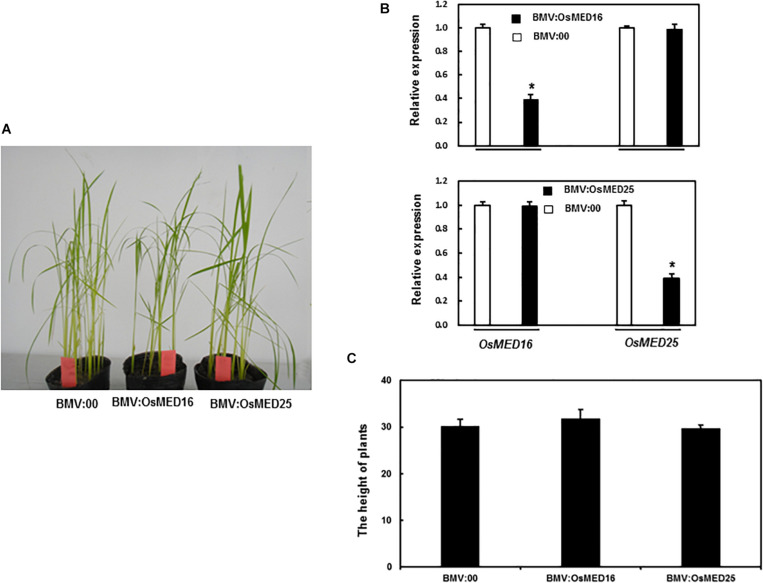
The growth condition of BMV:OsMED16- and BMV:OsMED25- infiltrated plants. **(A)** Growth conditions of BMV: OsMED16-, BMV: OsMED25-, and BMV:00-infiltrated plants. **(B)** The silencing specificity of *OsMED16* and *OsMED25* in BMV:OsMED16- or BMV:OsMED25-infiltrated plants. 10-day-old IR64 plants were infiltrated with agrobacteria carrying BMV: OsMED16-, BMV:OsMED25 or BMV:00 constructs and leaf samples were collected at 4 weeks after agroinfiltration. Transcript levels for *OsMED16* and *OsMED25* were analyzed by qRT-PCR using a rice *OsActin* gene as an internal control. **(C)** Heights of BMV: OsMED16-, BMV: OsMED25-, and BMV:00-infiltrated plants. *Above the columns indicate significant differences at *p* < 0.05 between the silencing plants and the control.

To better understand the biological functions of *OsMED16* and *OsMED25*, we generated BMV:OsMED16- and BMV:OsMED25-infiltrated seedlings by VIGS. The silencing efficiency of the target gene was assessed by qRT-PCR. As shown in Figure 5B, the silencing efficiency for these two genes was approximately 60%. The silenced seedlings were selected for further experiments. The BMV:OsMED16- and BMV:OsMED25-infiltrated seedlings grew and developed normally during our observation as compared with BMV:00-infiltrated plants (Figures 5A,C). We believe that *OsMED16* or *OsMED25* silencing did not affect the growth and development of rice seedlings.

### *OsMED16* and *OsMED25* Silencing Decreased the Resistance to *M. grisea*

**FIGURE 6:**
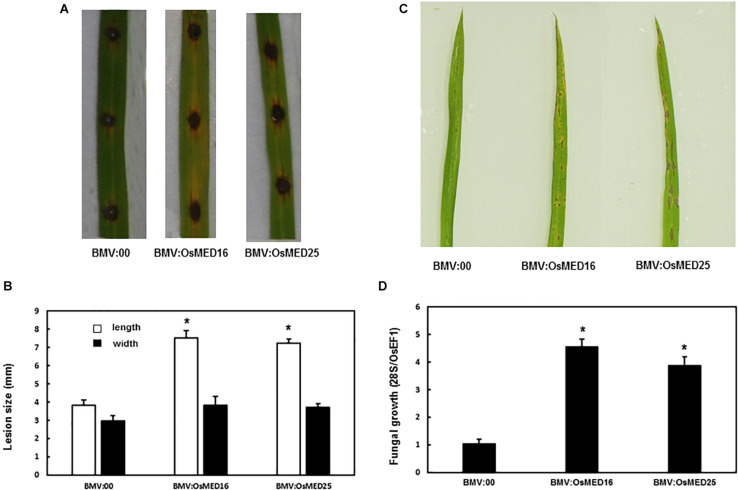
BMV:OsMED16- and BMV:OsMED25-infiltrated plants displayed reduced resistance to *Magnaporthe grisea* when compared with BMV:00-infiltrated plants. **(A)** The phenotype of BMV: OsMED16-, BMV: OsMED25-, and BMV:00-infiltrated plants following *M. grisea* inoculation by detached leaf analysis. **(B)** The lesion size was measured using the detached leaf analysis. **(C)** The phenotype of BMV: OsMED16-, BMV: OsMED25-, and BMV:00-infiltrated plants following *M. grisea* inoculation by whole plant analysis. **(D)** Fungal growth in BMV: OsMED16-, BMV: OsMED25-, and BMV:00-infiltrated plants following *M. grisea* inoculation. *Above the columns indicate significant differences at *p* < 0.05 between the silencing plants and the control.

*Magnaporthe grisea* was inoculated into the detached leaves and whole plants following the above-described method. The detached leaf assay revealed that the lesions on the leaves of BMV:OsMED16- and BMV:OsMED25-infiltrated plants were considerably larger than those on BMV:00-infiltrated plants (Figures 6A,B). The whole plant analysis showed similar results. The disease phenotype of BMV:OsMED16- or BMV:OsMED25-infiltrated plants was severe than that of BMV:00-infiltrated plants (Figure 6C) with more fungal growth (Figure 6D). This implied that compared with BMV:00-infiltrated plants, BMV:OsMED16- and BMV:OsMED25-infiltrated plants increased the susceptibility to *M. grisea*.

**FIGURE 7:**
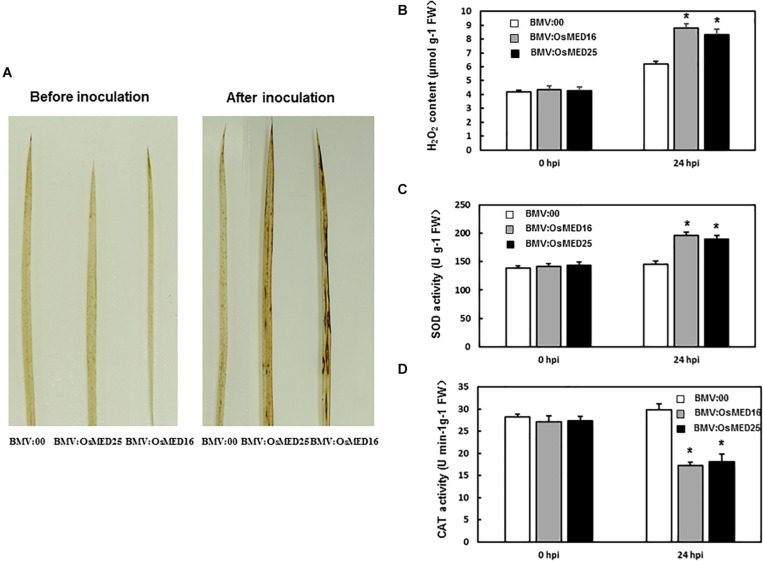
The H_2_O_2_ condition in BMV: OsMED16-, BMV: OsMED25-, and BMV:00-infiltrated plants before and after *Magnaporthe grisea* inoculation. **(A)** The DAB staining of leaves from BMV: OsMED16-, BMV: OsMED25-, and BMV:00-infiltrated plants before and after *M. grisea* inoculation. **(B)** The H_2_O_2_ content in BMV: OsMED16-, BMV: OsMED25-, and BMV:00-infiltrated plants before and after *M. grisea* inoculation. **(C)** The SOD activity in BMV: OsMED16-, BMV: OsMED25-, and BMV:00-infiltrated plants before and after *M. grisea* inoculation. **(D)** The CAT activity in BMV: OsMED16-, BMV: OsMED25-, and BMV:00-infiltrated plants before and after *M. grisea* inoculation. *Above the columns indicate significant differences at *p* < 0.05 between the silencing plants and the control.

To explore the mechanism for reduced resistance to *M. grisea* following *OsMED16* and *OsMED25* silencing, H_2_O_2_ content and the expression patterns of defense-related genes were analyzed. First, H_2_O_2_ condition was analyzed. DAB staining results showed that BMV:OsMED16- and BMV:OsMED25-infiltrated plants accumulated more H_2_O_2_ than BMV:00-infiltrated plants following the inoculation with *M. grisea* (Figure 7A). However, there were no significant differences in H_2_O_2_ accumulation in BMV: OsMED16-, BMV: OsMED25-, and BMV:00-infiltrated plants without the inoculation of *M. grisea*. This finding was further confirmed by quantification of H_2_O_2_ concentrations (Figure 7B). The SOD activity in BMV:OsMED16- and BMV:OsMED25-infiltrated plants was higher than that in BMV:00-infiltrated plants after the inoculation of *M. grisea*, whereas the CAT activity was lower (Figures 7C,D).

**FIGURE 8:**
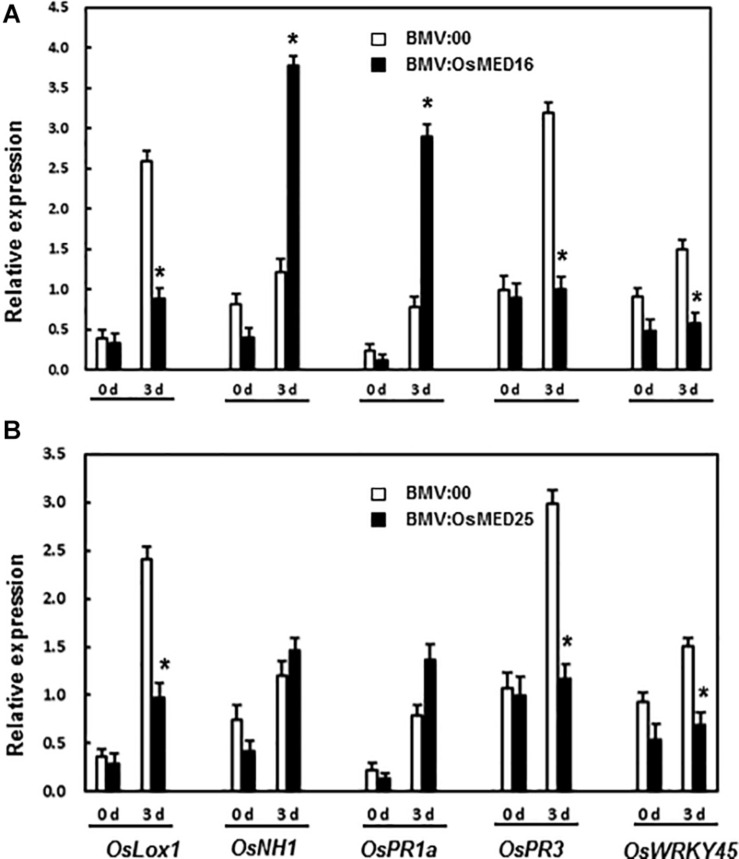
The expression pattern of defense-related genes in BMV:OsMED16- **(A)** and BMV:OsMED25-infiltrated plants **(B)** before and after *Magnaporthe grisea* inoculation. The expression of defense related genes was analyzed by qRT-PCR. Expression data were normalized against *OsActin* gene, and relative expression is shown as fold expression of *OsActin*. Data are presented as means ± SD from three independent experiments and * above the columns indicate significant differences at *p* < 0.05 between the silenced plants and the control.

Next, the expression of defense-related genes was analyzed. There existed no significant difference in the expression of *OsLOX1*, *OsNH1*, *OsPR1a*, *OsPR3*, and *OsWRKY45* between BMV:target genes- and BMV:00-infiltrated plants before the inoculation of *M. grisea*. Three days after the inoculation of *M. grisea*, the expression levels of *OsNH1* and *OsPR1a* in BMV:OsMED16-infiltrated plants increased significantly, whereas that of *OsLOX1*, *OsPR3*, and *OsWRKY45* decreased when compared BMV:00-infiltrated plants (Figure 8A). After the inoculation of *M. grisea*, the expression of *OsLOX1*, *OsPR3*, and *OsWRKY45* decreased in BMV:OsMED25-infiltrated plants, whereas that of *OsNH1* and *OsPR1a* remained unchanged when compared with that in BMV:00-infiltrated plants (Figure 8B).

### BMV:OsMED16-Infiltrated Plants Showed Decreased Tolerance to Cold

**FIGURE 9:**
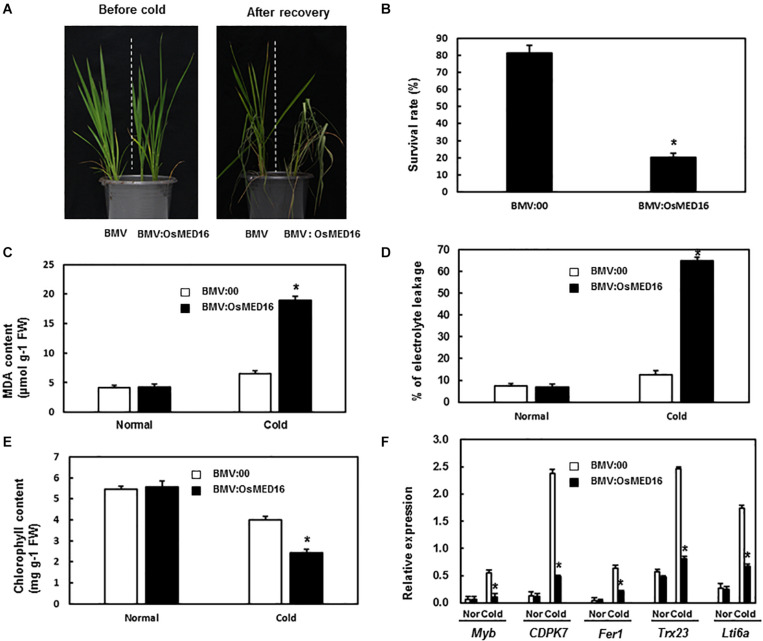
BMV:OsMED16-infiltrated plants showed decreased tolerance to cold stress when compared with BMV:00-infiltrated plants. **(A)** The phenotype of BMV:OsMED16- and BMV:00-infiltrated plants under cold stress. **(B)** The survival rate of BMV:OsMED16- and BMV:00-infiltrated plants after cold stress. **(C)** The MDA content in BMV:OsMED16- and BMV:00-infiltrated plants with and without cold stress. **(D)** The relative electrolyte leakage in BMV:OsMED16- and BMV:00-infiltrated plants with and without cold stress. **(E)** The chlorophyll content in BMV:OsMED16- and BMV:00-infiltrated plants with and without cold stress. **(F)** The expression of cold-responsive genes in BMV:OsMED16- and BMV:00-infiltrated plants before and after cold stress. *Above the columns indicate significant differences at *p* < 0.05 between the silencing plants and the control.

To study whether *OsMED16* or *OsMED25* contributed to the response to cold stress, the tolerance of BMV:OsMED16- and BMV:OsMED25-infiltrated plants to cold was assessed. The 4-week-old BMV:target genes- and BMV:00-infiltrated plants in the same pot were placed in the incubator at 4°C with a 14 h/10 h light/dark cycle. After 24 h, the plants were transferred to normal growth conditions for recovery. As shown in Figure 9A, less number of BMV:OsMED16-infiltrated plants were recovered from cold stress when compared with BMV:00-infiltrated plants. The survival rate of BMV:OsMED16-infiltrated plants was approximately 25% of BMV:00-infiltrated plants (Figure 9B).

To find out the possible mechanism of *OsMED16* silencing resulting in a decreased tolerance to cold, the MDA content, relative electrolyte leakage, chlorophyll content, and the expression of cold-responsive genes in BMV:OsMED16- and BMV:00-infiltrated plants under unstressed or cold stress were analyzed. The MDA content and relative electrolyte leakage in BMV:OsMED16-infiltrated plants increased dramatically after cold stress when compared with BMV:00-infiltrated plants, whereas there existed no significant difference under unstressed conditions (Figures 9C,D). The chlorophyll content in BMV:OsMED16-infiltrated plants decreased dramatically after cold stress when compared with BMV:00-infiltrated plants (Figure 9E). Finally, the expression of cold-responsive genes was measured in BMV:OsMED16- and BMV:00-infiltrated plants. As shown in Figure 9F, the expression of *Myb*, *CDPK7*, *Fer1*, *Trx23*, and *Lti6a* decreased dramatically in BMV:OsMED16-infiltrated plants when compared with BMV:00-infiltrated plants under cold stress.

## Discussion

The plant mediator complex was first purified from *Arabidopsis* in 2007 (Backstrom et al., 2007). Since then, several studies have been conducted on its functions in plants. The complex has been implicated in several processes, such as development, abiotic and biotic stress responses, and many other cellular activities, including on-encoding RNA functions, regulation of DNA and protein stability, and secondary metabolism (Kidd et al., 2011; Kim and Chen, 2011; Kim et al., 2011; Bonawitz et al., 2014; Gillmor et al., 2014; Hemsley et al., 2014; Raya-González et al., 2014; Yang et al., 2014; Zhang et al., 2014; Li et al., 2015). The majority of the work has been performed in *Arabidopsis*, with only a few studies in rice. In our present study, the functions of *OsMED16* and *OsMED25* were studied in rice.

The expression of the mediator complex is induced by pathogen inoculation and exposure to stresses (Hussein et al., 2020; Nath et al., 2020). For instance, the transcription of ScMED7 is induced by heavy metals (CdCl_2_), low temperatures (4°C), and hormones (SA and MeJA), whereas it is inhibited by osmotic stresses by NaCl and PEG (Zhang et al., 2017). The expression of MED16 is induced by *Pseudomonas syringae*, ultraviolet-C (UV-C) irradiation, SA, and JA (Wathugala et al., 2012). In our study, the expression of *OsMED16* and *OsMED25* was induced by the inoculation of rice plants with *M. grisea*, hormone treatment, and abiotic stresses (Figures 2, 3). The results showed different expression patterns implying varying functions in response to biotic and abiotic stresses. The expression of *OsMED14* was high in roots, leaves, anthers, and seeds at younger stages (Malik et al., 2020). Although ScMED7 is constitutively expressed, the expression is significantly higher in bud tissues. Furthermore, in our study, *OsMED16* and *OsMED25* were constitutively expressed in all tissues we studied, however, with different patterns (Figure 4).

The mediator complex was reported to be involved in development. MED25 exerts effects on organ size, root hair differentiation, and root system architecture (Xu and Li, 2011; Sundaravelpandian et al., 2013a,b; Raya-González et al., 2014). However, no significant difference was found between the root of BMV:OsMED25-infiltrated plants and those of BMV:00-infiltrated plants (data not shown). The mutation in MED17, MED18, or MED20 resulted in dwarfism, delayed flowering, and reduced fertility (Kim et al., 2011). However, we did not observe any significant difference in growth and development between BMV:target gene- and BMV:00-infiltrated plants (Figure 5). We speculated that *OsMED16* and *OsMED25* did not function in the vegetative stage. Whether *OsMED16* and *OsMED25* have a function in the reproductive stage needs to be further studied.

The mediator complex also functions in plant immunity (Canet et al., 2012; Cevik et al., 2012; Chen et al., 2012; Caillaud et al., 2013; Zhang et al., 2013). For example, MED16 mutations in *Arabidopsis* decreased the resistance to *Pst* DC3000/avrRpt2, *Pst* DC3000, *A. brassicicola*, and *B. cinerea*. MED16 could be a positive regulator of SAR, regulating the responsive capability of SA and the basal resistance (Wathugala et al., 2012; Zhang et al., 2012). The med25 mutants decreased the resistance to *A. brassicicola* and *B. cinerea* and increased the resistance to *F. oxysporum* (Kidd et al., 2009). OsMED16 and OsMED25 showed sequence similarity to AtMED16 and AtMED25, respectively (Figure 1). Therefore, we studied whether *OsMED16* and *OsMED25* functioned in plant immunity in rice and our results indicated that the silencing of *OsMED16* and *OsMED25* reduced the resistance to *M. grisea*, produced larger lesions, and increased fungal growth (Figure 6).

The relationship between plant immunity and ROS generation is well described. The ROS accumulation is beneficial for pathogen infection. The function of H_2_O_2_, a kind of ROS, in disease resistance, has gained extensive attention. H_2_O_2_ accumulation was found in several plant-pathogen systems. We first analyzed H_2_O_2_ conditions to explore the mechanism of reduced resistance caused by the silencing of *OsMED16* and *OsMED25*. As shown in Figure 7A, BMV:OsMED16- and BMV:OsMED25-infiltrated plants accumulated more H_2_O_2_ than BMV:00-infiltrated plants following *M. grisea* inoculation, which was confirmed by H_2_O_2_ quantification (Figure 7B). The SOD and CAT activities were analyzed to explain the reason for elevated H_2_O_2_ levels. SOD catalyzes superoxide anions to H_2_O_2_ and O_2_, and CAT catalyzes H_2_O_2_ to H_2_O and O_2_. Higher SOD activity in BMV:OsMED16- and BMV:OsMED25-infiltrated plants implied more H_2_O_2_ production, whereas a lower CAT activity in BMV:OsMED16- and BMV:OsMED25-infiltrated plants meant less H_2_O_2_ consumption (Figures 7C,D). This could explain the high H_2_O_2_ content in BMV:OsMED16- and BMV:OsMED25-infiltrated plants.

Next, the expression of defense-related genes in BMV: OsMED16-, BMV: OsMED25-, and BMV:00-infiltrated plants before and after *M. grisea* inoculation was analyzed to explore the mechanism of decreased resistance caused by the silencing of *OsMED16* and *OsMED25*. The expression of both *OsNH1* and *OsPR1a* increased dramatically in BMV:OsMED16-infiltrated plants after the infection of *M. grisea* when compared with BMV:00-infiltrated plants, whereas the expression of *OsLOX1*, *OsPR3*, and *OsWRKY45* decreased (Figure 8A). This result indicated that the expression of JA-responsive genes decreased, whereas SA-responsive genes increased in BMV:OsMED16-infiltrated plants after the infection of *M. grisea*. And OswWRKY45 is a positive regulator of resistance to fungal pathogens. As reported previously, MED25 is a positive regulator of the expression of JA-responsive genes. The expression of several defense genes, especially JA-responsive genes, was decreased dramatically in *med25* mutants, whereas that of SA-responsive genes showed no dramatic difference. The mutants showed no difference in the resistance to *Pst*DC3000 and SAR induced by organisms compared with the control (Kidd et al., 2009). In our study, the expression of JA-responsive genes (such as *OsLOX1* and *OsPR3*) decreased, whereas that of SA-responsive genes (such as *OsNH1* and *OsPR1a*) remained unchanged in BMV:OsMED25-infiltrated plants after the infection of *M. grisea* when compared with BMV:00-infiltrated plants (Figure 8B).

MED25 that interacts with DREB2A, ZFHD1, and Myb transcription factor regulates the response to abiotic stresses. Its mutants were more sensitive to salt stress with reduced resistance to drought (Elfving et al., 2011). However, BMV:OsMED25-infiltrated seedlings showed no difference from BMV:00-infiltrated seedlings in response to abiotic stresses. MED16, along with MED2 and MED14, regulates cold stress and its mutants decreased the resistance to cold and osmotic stress (Boyce et al., 2003; Knight et al., 2009; Hemsley et al., 2014). Moreover, we observed that the BMV:OsMED16-infiltrated plants displayed reduced resistance to cold when compared with the control (Figures 9A,B).

Under abiotic stresses, plants produce excessive radicals causing peroxidation injury, metabolic disorders, and antioxidant imbalance. Plants have evolved enzymatic and non-enzymatic systems to scavenge ROS and maintain the balance between ROS generation and ROS scavenging ability. Reduced activity of scavenging enzymes increases ROS species, including peroxides and MDA. MDA content is often considered as a physiological index for the degree of cell membrane damage and lipid peroxidation. High levels of MDA are toxic to plant cells (You and Chan, 2015). Furthermore, relative electrolyte leakage is an important index to judge the extent of damage after abiotic stresses. The extent of damage to the plants increases with the severity of plant stresses. Chlorophyll content partly implies the growth condition of plants. In contrast, the expression of cold-responsive genes was one of the contributing factors to cold tolerance. Therefore, the MDA content, relative electrolyte leakage, chlorophyll content, and the expression of cold-responsive genes were analyzed to explore the mechanism of decreased tolerance to cold stress caused by *OsMED16* silencing. In our study, the MDA content and relative electrolyte leakage in BMV:OsMED16-infiltrated seedlings increased notably when compared with the BMV:00-infiltrated seedlings, whereas the chlorophyll content decreased (Figures 9C–E). The expression of cold-responsive genes downregulated in BMV:OsMED16-infiltrated seedlings when compared with BMV:00-infiltrated seedlings (Figure 9F). These results showed that BMV:OsMED16-infiltrated seedlings reduced the tolerance to cold stress through increased MDA content and decreased expression of cold-responsive genes.

Altogether, *OsMED16* and *OsMED25* contribute to the response to biotic stresses, probably by regulating the H_2_O_2_ content and the expression of defense-related genes. BMV:OsMED16-infiltrated plants displayed decreased tolerance to cold stress, probably by regulating MAD content and the expression of cold-responsive genes.

## Data Availability Statement

The raw data supporting the conclusions of this article will be made available by the authors, without undue reservation.

## Author Contributions

HZ, DZ, LY, MJ, and FS carried out most of the experiments. MJ and HZ designed the experiments and wrote the manuscript. All authors read and approved the final manuscript.

## Conflict of Interest

The authors declare that the research was conducted in the absence of any commercial or financial relationships that could be construed as a potential conflict of interest.
