# Supplementary figures and images for: Functional Analysis of OsMED16 and OsMED25 in Response to Biotic and Abiotic Stresses in Rice
Source: Front Plant Sci. 2021 Mar 31;12:652453. doi: 10.3389/fpls.2021.652453 (PMC8044553; doi:10.3389/fpls.2021.652453)

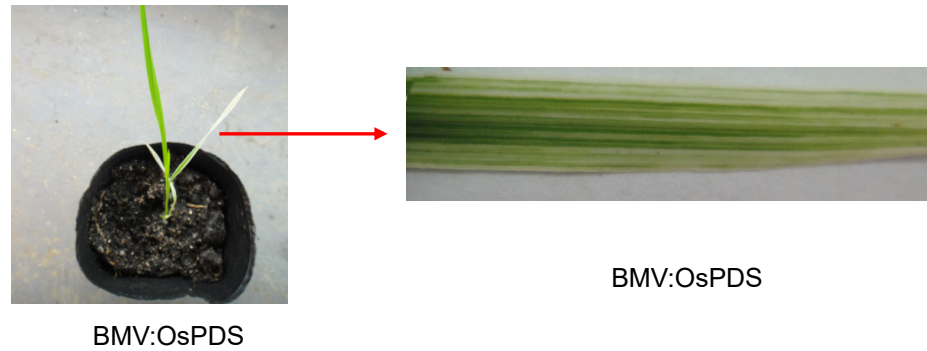

**Figure 1 The image of the BMV:OsPDS**

Supplement: Supplementary file 1 [file Image_1.pdf]
