# Supplementary material for: Functional Analysis of OsMED16 and OsMED25 in Response to Biotic and Abiotic Stresses in Rice
Source: Front Plant Sci. 2021 Mar 31;12:652453. doi: 10.3389/fpls.2021.652453 (PMC8044553; doi:10.3389/fpls.2021.652453)
Supplement: Supplementary file 2 [file Data_Sheet_1.docx]

The sequence of *OsMED16* fragment used in VIGS was:

TGTCAGGAATTTCTCCGTATAGAT GGCTTCCTGCAAACTCTAGTACTTCATC AAACTTGAAAACCTTTGAGGAAAAGTTCCTTACCCAGCAGCCTCAAAGTTCGGCTGGGTGGCCAAACATTCTATGTGTCTGTTCAGTTTTTTCATCGGGTTCTGTTCAGCTTCATTGGTCACAATGGCCTTCTCAAAACTCAGCACAACCTAGATGGTTTTCTACTAGCAAAGGGCTTTTAGGAGCAGGGCCAAGCGGCATAATGGCTGCTGATGCTATTATTACTGAAACTGGAGCATTACATGTTGCTGGTGTTCCCCTTGTTAATCCATCTACTGTAGTGGTTTGGGAGGTGA

The sequence of *OsMED25* fragment used in VIGS was: ATACAATGCGGCAAAGAGGAATCCTCGAGCGGCTGACCC ATCAGTGGATC ATGCAAAAAATCCACATTTTCTTGTTTTGTTGTCTGACAATTTTTTGGAGGCTCGAACTGCTCTAAGTCGCCCTTTACCTGGCAACTTGGTCACAAATCACCCCATTACAAAAATGGATACAGCTGCAACATCTGTGCCAGTACCAACTTCAAATGGCAACCCCTCAGTTAATGGACCTATGCTTACCCGCCAACCAAATGGTGTTGTTGCAAATATTAAAACGGAGCCAACAACTTTACCGCCCATGGTTTCTGCACCTGCTTTCTCGCATGTAACACCTGTTGCAAATGGTGTTTCACAAGGATTATCATCAGTACAAAGTCCCTCACCGTCCCTTATTT
